# Avalanche of entanglement and correlations at quantum phase transitions

**DOI:** 10.1038/s41598-017-03402-8

**Published:** 2017-06-16

**Authors:** Konstantin V. Krutitsky, Andreas Osterloh, Ralf Schützhold

**Affiliations:** Institut für Theoretische Physik, Universität Duisburg-Essen, D-47048 Duisburg, Germany

## Abstract

We study the ground-state entanglement in the quantum Ising model with nearest neighbor ferromagnetic coupling *J* and find a sequential increase of entanglement depth *d* with growing *J*. This entanglement avalanche starts with two-point entanglement, as measured by the concurrence, and continues via the three-tangle and four-tangle, until finally, deep in the ferromagnetic phase for *J* = ∞, arriving at a pure *L*-partite (GHZ type) entanglement of all *L* spins. Comparison with the two, three, and four-point correlations reveals a similar sequence and shows strong ties to the above entanglement measures for small *J*. However, we also find a partial inversion of the hierarchy, where the four-point correlation exceeds the three- and two-point correlations, well before the critical point is reached. Qualitatively similar behavior is also found for the Bose-Hubbard model, suggesting that this is a general feature of a quantum phase transition. This should be taken into account in the approximations starting from a mean-field limit.

## Introduction

Entanglement is one of the main reasons for the complexity of quantum many-body systems: the more entangled a quantum system is the more complex its description becomes. If we consider e.g. ground states of lattice Hamiltonians and have no entanglement between the lattice sites *i*, the quantum state is fully separable $|{\rm{\Psi }}\rangle ={\otimes }_{i}|{\psi }_{i}\rangle$. As a result, it is possible to employ a mean-field description where observables ${\hat{A}}_{i}$ and ${\hat{B}}_{j}$ at different lattice sites *i* and *j* are uncorrelated $\langle {\hat{A}}_{i}{\hat{B}}_{j}\rangle =\langle {\hat{A}}_{i}\rangle \langle {\hat{B}}_{j}\rangle$. However, except for very few special cases (e.g., Kurmann-Thomas-Müller point^1, 2^), such a description is not exact. It is possible to improve this mean-field ansatz by adding some amount of entanglement. This can be achieved either directly^3^ or with matrix product states^4, 5^ or tree-tensor networks^6–8^. These descriptions make sense only if the entanglement is bounded in a suitable way as highlighted in ref. 9. However, one should be aware that this criterion does not apply in most relevant cases, where the reduced density matrix is of full rank, even though some of the eigenvalues may be small. Thus, its application requires approximations such as (2).

On the other hand, many interesting phenomena in condensed matter are associated with and occur at or close to quantum critical points, where typically the entanglement becomes relevant. As one of the simplest yet prototypical examples^10^, let us consider the one-dimensional Ising model of length *L* in a transverse field

1 $$\hat{H}=-J\sum _{i=1}^{L}\,{\hat{\sigma }}_{i}^{z}{\hat{\sigma }}_{i+1}^{z}-\sum _{i=1}^{L}\,{\hat{\sigma }}_{i}^{x},$$

where ${\hat{\sigma }}_{i}^{x,y,z}$ denote the spin-1/2 Pauli matrices acting on the lattice site *i* and periodic boundary conditions ${\sigma }_{L+1}^{\alpha }={\sigma }_{1}^{\alpha },\alpha =x,y,z,$ are imposed. For *L* → ∞, we have a symmetry-breaking second-order quantum phase transition from the paramagnetic phase at |*J*| < 1 to ferromagnetism at *J* > 1^10^. For *J* = 0, we have the separable paramagnetic state $|\to \to \to \ldots \rangle$ without entanglement while for *J* → ∞, the ground state corresponds to the ferromagnetic state $(|\uparrow \uparrow \uparrow \ldots \rangle +|\downarrow \downarrow \downarrow \ldots \rangle )/\sqrt{2}$ with GHZ-type multi-partite entanglement between all *L* spins. We want to mention that this GHZ state, however, would not survive small added field in the (*y*, *z*) plane. Here, |→〉 is the eigenstate of *σ* ^*x*^ and $|\uparrow \rangle$ that of *σ* ^*z*^ to the eigenvalue +1. At the critical point *J*_crit_ = 1, the entanglement entropy between the left and the right half of the Ising chain diverges as ln *L* ^11^.

**Figure 1:**
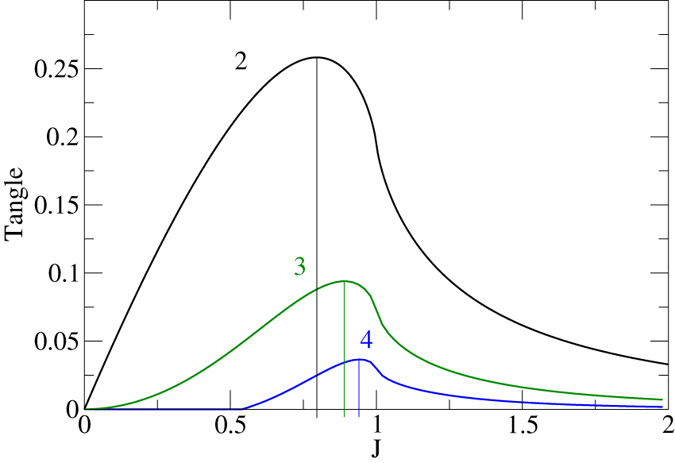
The entanglement between two, three, and four neighboring spins measured by the concurrence *C*_2_ (black), the three-tangle $\sqrt{{\tau }_{3}}$ (green), and the four-tangle *τ*_4_ (blue) as a function of *J*. For the latter two, the approximation (2) was used. They take their maximum values at ${J}_{2}^{{\rm{\max }}}\approx 0.796$, ${J}_{3}^{{\rm{\max }}}\approx 0.890$, and ${J}_{4}^{{\rm{\max }}}\approx 0.94$, respectively – which shows the sequential increase of entanglement depth (avalanche of entanglement).

However, this large amount of entanglement cannot be explained by entanglement of pairs alone^12^, as measured by the concurrence. Together with the entanglement monogamy relation^13, 14^ (see also refs 15–18) this strongly suggests the emergence of multipartite entanglement^19, 20^ (triples and quadruples etc.), which will be studied in the following section (see the results in Fig. 1). Multipartite quantum correlations will be analyzed later on, also with reference to another prototypical model of quantum phase transitions, the Bose-Hubbard model.

## Entanglement

In order to study the multipartite entanglement during the quantum phase transition of the Ising model, we employ its exact solution via Jordan-Wigner and Bogoliubov transformation to a free fermionic model^21, 22^ which allows us to obtain the reduced density matrices of two ${\hat{\rho }}_{2}={\hat{\rho }}_{ij}$, three ${\hat{\rho }}_{3}={\hat{\rho }}_{ijk}$, and four ${\hat{\rho }}_{4}={\hat{\rho }}_{ijkl}$ neighboring spins^19, 21^. After diagonalizing these matrices, we find that they all possess two dominant eigenvalues *p*_1_ and *p*_2_ while the sum of the remaining sub-dominant eigenvalues stays below 2.5% (see supplementary information). Thus, we approximate the two-point reduced density operators as

2 $${\hat{\rho }}_{ij}\approx {p}_{1}|{\psi }_{ij}^{1}\rangle \langle {\psi }_{ij}^{1}|+(1-{p}_{1})|{\psi }_{ij}^{2}\rangle \langle {\psi }_{ij}^{2}|,$$

and analogously for ${\hat{\rho }}_{ijk}$ and ${\hat{\rho }}_{ijkl}$. Actually, the accuracy of this approximation should be even better than 2.5% in the weights of the density matrix: while the multi-partite entanglement of the first $|{\psi }_{\ldots }^{1}\rangle$ and the second $|{\psi }_{\ldots }^{2}\rangle$ eigenvectors can interfere destructively with each other, we checked that this is not the case for the third eigenvector $|{\psi }_{\ldots }^{3}\rangle$ which has a different structure: for three and four spins, the state(s) of the central spin(s) are fixed to |→〉 while the two boundary spins form a Bell state – i.e., $|{\psi }_{\ldots }^{3}\rangle$ contains bipartite entanglement only, which here does not interfere with the multipartite entanglement of $|{\psi }_{\ldots }^{1}\rangle$ and $|{\psi }_{\ldots }^{2}\rangle$. As a result, we expect that the accuracy of this approximation is around 0.5% or even better. Entanglement measures scale like $\sqrt{p}$ since this is the weight of how the states are added^23^; therefore, this corresponds to an error of about 7%. For two spins, we checked this approximation by comparing the exact concurrence with that derived from (2) and found that they are virtually indistinguishable^24^. The approximation (2) as motivated by the dominance of the two largest eigenvalues is a great simplification, because we obtain rank-two density matrices, for which the three-tangle *τ*_3_ and the four-tangle(s) *τ*_4_ (see Methods) can be calculated exactly^23, 25^ for this model. Note that for the three-tangle an exact extension to arbitrary mixed states by the convex roof is not known so far.

**Figure 2:**
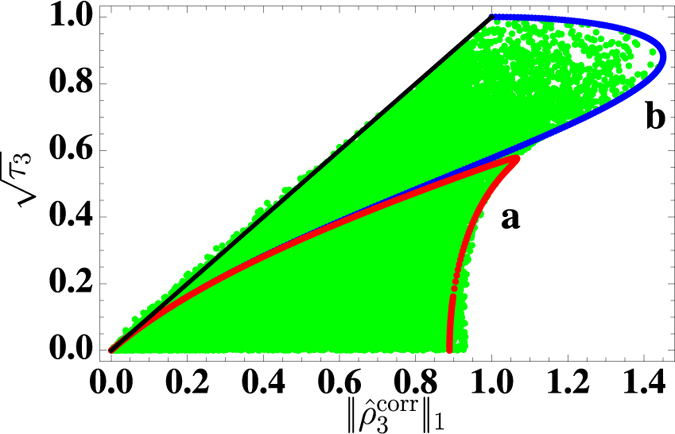
Plot of the three-tangle $\sqrt{{\tau }_{3}}$ versus the three-point correlation bound ${\Vert {\rho }_{3}^{{\rm{corr}}}\Vert }_{1}$ for a random selection of 40.000 pure states (4) from the generalized Schmidt decomposition^34, 35^. Each dot in the figure corresponds to a single state. The thin black line corresponds to $\sqrt{{\tau }_{3}}={\Vert {\hat{\rho }}_{3}^{{\rm{corr}}}\Vert }_{1}$. Most of the states satisfy the inequality ${\Vert {\rho }_{3}^{{\rm{corr}}}\Vert }_{1}\ge \sqrt{{\tau }_{3}}$ (as in the case of two spins where ${\Vert {\rho }_{2}^{{\rm{corr}}}\Vert }_{1}\ge {C}_{2}$ is valid for all pure states) but some of them slightly deviate from it. The lower red (a) and upper blue curve (b) are obtained for GHZ states that are made of two $(\alpha |\uparrow \uparrow \uparrow \rangle +\beta |\downarrow \downarrow \downarrow \rangle )$ and four $(\alpha |\uparrow \uparrow \uparrow \rangle +\beta |{\rm{W}}\rangle )$ product basis elements, respectively, where $|{\rm{W}}\rangle =(|\downarrow \uparrow \uparrow \rangle +|\uparrow \downarrow \uparrow \rangle +|\uparrow \uparrow \downarrow \rangle )/\sqrt{3}$.

In analogy to the three-tangle *τ*_3_, we call four-tangles those polynomial *SL*-invariants that are zero for arbitrary product states^26^ and use the notation ${\tau }_{4}^{(i)}$, *i* = 1, 2, 3, for powers that scale linearly in the density matrix. All three of them essentially lead to the same output, and therefore *τ*_4_ will represent the four-partite entanglement content of the model. This four-partite entanglement would hence be essentially of GHZ-type because only the GHZ entanglement is measured by all three measures in the same way^26–28^. Together with the results from ref. 29 this is an evidence for GHZ class entanglement also for four sites (for three sites see ref. 20). The quantifiers *τ*_3_ (for the tripartite entanglement in ${\hat{\rho }}_{3}$) and *τ*_4_ (for the quadripartite entanglement in ${\hat{\rho }}_{4}$) are shown in Fig. 1 for nearest neighbors. For various reasons, it is advantageous to consider $\sqrt{{\tau }_{3}}$ instead of *τ*_3_. First, this quantity $\sqrt{{\tau }_{3}}$ is an entanglement monotone^30^. Second, it is a homogeneous function of degree one in the density matrix ${\hat{\rho }}_{3}$, as the other measures *C*_2_ and *τ*_4_ considered here – or more general measures with the properties of probabilities. Third, this power $\sqrt{{\tau }_{3}}$ facilitates a direct comparison to the correlations, see Fig. 2. For completeness, we also included the pairwise entanglement of nearest neighbors, as measured by the concurrence *C*_2_.

As is well-known, the concurrence first grows as a function of *J* until it reaches a maximum at $J\approx 0.796$ and later decreases again (with an infinite slope at the critical point^31^). The three-tangle $\sqrt{{\tau }_{3}}$ starts to grow much slower at small *J* and reaches its maximum later than the concurrence at $J\approx 0.89$. The four-tangle(s) *τ*_4_ are even zero until $J\approx 0.55$ and reach their maximum yet a bit later at $J\approx 0.94$. Even though having no results for more spins, we conjecture that this sequence or avalanche of entanglement continues until finally, deep in the ferromagnetic phase, we get pure *L*-partite entanglement of all spins. This is clear since we have the *L*-particle GHZ-state as a ground state of the Hamiltonian. The GHZ-state is a stochastic state (local density matrix is proportional to {\mathbb{1}}) connected to a minimal length of 2 (see ref. 32) and therefore will maximize at least one (virtually all) SL-invariant homogenous polynomial measure of entanglement^30^. This feature survives in the odd sector of the symmetry group ${{\mathbb{Z}}}_{2}$ of the transverse Ising model.

## Entanglement versus correlations

As already mentioned before, a pure state without any entanglement is fully separable $|{\rm{\Psi }}\rangle ={\otimes }_{i}|{\psi }_{i}\rangle$ and observables ${\hat{A}}_{i}$ and ${\hat{B}}_{j}$ at different lattice sites *i* and *j* are uncorrelated. In the following, we shall study the relation between entanglement and the resulting correlations for more general states. To this end, we start with the reduced density matrices for one ${\hat{\rho }}_{1}={\hat{\rho }}_{i}$, two ${\hat{\rho }}_{2}={\hat{\rho }}_{ij}$, three ${\hat{\rho }}_{3}={\hat{\rho }}_{ijk}$, and four ${\hat{\rho }}_{4}={\hat{\rho }}_{ijkl}$ spins and separate correlated parts via ${\hat{\rho }}_{ij}^{{\rm{corr}}}={\hat{\rho }}_{ij}-{\hat{\rho }}_{i}{\hat{\rho }}_{j}$, and analogously for more spins (see Methods). The correlation between the two observables ${\langle {\hat{A}}_{i}{\hat{B}}_{j}\rangle }^{{\rm{corr}}}=\langle {\hat{A}}_{i}{\hat{B}}_{j}\rangle -\langle {\hat{A}}_{i}\rangle \langle {\hat{B}}_{j}\rangle$ can be written as ${\langle {\hat{A}}_{i}{\hat{B}}_{j}\rangle }^{{\rm{corr}}}={\rm{Tr}}\{{\hat{A}}_{i}{\hat{B}}_{j}{\hat{\rho }}_{ij}^{{\rm{corr}}}\}$ and similarly for three or more sites.

Since correlations such as ${\langle {\hat{A}}_{i}{\hat{B}}_{j}\rangle }^{{\rm{corr}}}$ and ${\langle {\hat{A}}_{i}{\hat{B}}_{j}{\hat{C}}_{k}\rangle }^{{\rm{corr}}}$ obviously depend on the observables ${\hat{A}}_{i}$, ${\hat{B}}_{j}$, and ${\hat{C}}_{k}$, it is convenient to derive an estimate directly from the correlated density matrices such as ${\hat{\rho }}_{ij}^{{\rm{corr}}}$. For observables ${\hat{A}}_{i}$ and ${\hat{B}}_{j}$ (such as the Pauli spin matrices) whose eigenvalues squared are bounded by unity (spectral norm) ${\hat{A}}_{i}^{2}\le {{\mathbb{1}}}_{i}$ and ${\hat{B}}_{j}^{2}\le {{\mathbb{1}}}_{j}$ which implies for their expectation values $|\langle {\hat{A}}_{i}{\hat{B}}_{j}\rangle |\le 1$, we obtain

3 $$|{\langle {\hat{A}}_{i}{\hat{B}}_{j}\rangle }^{{\rm{corr}}}|=|{\rm{Tr}}\{{\hat{A}}_{i}{\hat{B}}_{j}{\hat{\rho }}_{ij}^{{\rm{corr}}}\}|=|\sum _{I}\,{\lambda }_{I}\langle {\chi }_{ij}^{I}|{\hat{A}}_{i}{\hat{B}}_{j}|{\chi }_{ij}^{I}\rangle |\le \sum _{I}\,|{\lambda }_{I}|={\Vert {\hat{\rho }}_{ij}^{{\rm{corr}}}\Vert }_{1},$$

where *λ*_*I*_ and $|{\chi }_{ij}^{I}\rangle$ are eigenvalues and eigenvectors of ${\hat{\rho }}_{ij}^{{\rm{corr}}}$. Equation (3) shows that the Schatten one-norm ${\Vert {\hat{\rho }}_{ij}^{{\rm{corr}}}\Vert }_{1}$ of the correlated density matrix ${\hat{\rho }}_{ij}^{{\rm{corr}}}$ yields an upper estimate for the correlations ${\langle {\hat{A}}_{i}{\hat{B}}_{j}\rangle }^{{\rm{corr}}}$ of all observables with ${\hat{A}}_{i}^{2}\le {{\mathbb{1}}}_{i}$ and ${\hat{B}}_{j}^{2}\le {{\mathbb{1}}}_{j}$. Obviously, this relation can be generalized to three or more sites in complete analogy. In the following, we shall focus on ${\Vert {\hat{\rho }}_{q}^{{\rm{corr}}}\Vert }_{1}$ for different number of sites *q*.

For two spins, it is well-known that the largest correlation function for pure states coincides with the concurrence^33^. For mixed states, this becomes an upper bound, i.e., the maximum correlation is larger or equal to the concurrence ${\Vert {\hat{\rho }}_{ij}^{{\rm{corr}}}\Vert }_{1}\ge {C}_{2}$. Unfortunately, for three or more spins, such a rigorous bound is not known. Thus, let us consider a system of three spins. A pure state of the system can be represented modulo local *SU*(2) transformations as the normalized superposition of five local basis product states^34, 35^

4 $$|{\psi }_{3}\rangle \cong A[{a}_{0}|000\rangle +{a}_{1}\,{e}^{i\phi }|100\rangle +{a}_{2}|101\rangle +{a}_{3}|110\rangle +{a}_{4}|111\rangle ],$$

with 0 ≤ *a*_*i*_ ≤ 1 and 0 ≤ *φ* < *π*. We generate the parameters *a*_*i*_ and *φ* from the corresponding uniform random distributions and choose *A* such that the resulting state is normalized. Then we calculate its three-tangle *τ*_3_ and ${\Vert {\hat{\rho }}_{3}^{{\rm{corr}}}\Vert }_{1}$. The results presented in Fig. 2 for 40.000 randomly chosen states $|{\psi }_{3}\rangle$ show that $\sqrt{{\tau }_{3}}$ is almost always upper bounded by ${\Vert {\hat{\rho }}_{3}^{{\rm{corr}}}\Vert }_{1}$. There are, however, some states for which $\sqrt{{\tau }_{3}}$ is slightly larger than ${\Vert {\hat{\rho }}_{3}^{{\rm{corr}}}\Vert }_{1}$. The reason for this slight deviation and the characteric properties of these states will be the subject of future investigations. Again we consider $\sqrt{{\tau }_{3}}$ and not *τ*_3_ because the former is a homogeneous function of degree one with respect to the density operator ${\hat{\rho }}_{3}$ and, therefore, has the same scaling properties as ${\Vert {\hat{\rho }}_{3}^{{\rm{corr}}}\Vert }_{1}$. Similar calculations for four spins indicate that ${\Vert {\hat{\rho }}_{ijkl}^{{\rm{corr}}}\Vert }_{1}$ is also only an approximate upper bound for the four-tangle *τ*_4_. However, since the available phase space for four spins is much larger, the statistics is rather poor^24^.

In summary, while the two-point correlation ${\hat{\rho }}_{ij}^{{\rm{corr}}}$ and the pairwise entanglement *C*_2_ are related via the *exact* bound ${\Vert {\hat{\rho }}_{ij}^{{\rm{corr}}}\Vert }_{1}\ge {C}_{2}$, we only find analogous approximate relations between the three- and four-point correlations ${\hat{\rho }}_{ijk}^{{\rm{corr}}}$ and ${\hat{\rho }}_{ijkl}^{{\rm{corr}}}$ on the one hand and the corresponding entanglement measures $\sqrt{{\tau }_{3}}$ and *τ*_4_ on the other hand.

## Correlations for the Ising model

**Figure 3:**
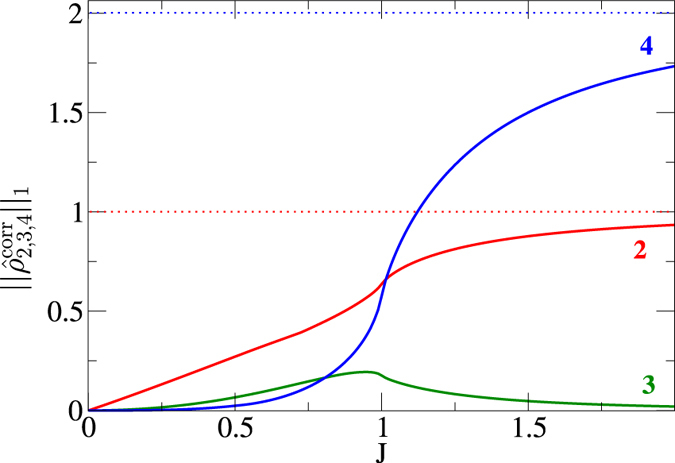
Norms of correlated reduced density operators for two ${\hat{\rho }}_{2}^{{\rm{corr}}}$ (red), three ${\hat{\rho }}_{3}^{{\rm{corr}}}$ (green), and four ${\hat{\rho }}_{4}^{{\rm{corr}}}$ (blue) neighboring spins in the transverse Ising model. At $J\approx 0.8$, i.e., well before the critical point, the 4-point correlations exceed the 3-point correlations. The 2-point correlations dominate both until the critical point is reached, afterwards the 4-point correlations prevail. The horizontal dashed lines represent the asymptotic values for *J* → ∞.

Motivated by the above findings, let us study the Schatten one-norms of the correlated density matrices for two, three, and four neighboring spins. Note that we used the exact results for the reduced density matrices without the approximation (2). The results are plotted in Fig. 3. As expected from stationary perturbation theory^24^, ${\Vert {\hat{\rho }}_{q}^{{\rm{corr}}}\Vert }_{1}\sim {J}^{q-1}$ for small *J*, where *q* = 2, 3, 4 is the number of the neighboring sites. Thus, for small *J*, the correlations obey the same hierarchy ${\Vert {\hat{\rho }}_{2}^{{\rm{corr}}}\Vert }_{1}\gg {\Vert {\hat{\rho }}_{3}^{{\rm{corr}}}\Vert }_{1}\gg {\Vert {\hat{\rho }}_{4}^{{\rm{corr}}}\Vert }_{1}$ as the entanglement measures in Fig. 1, except that ${\Vert {\hat{\rho }}_{4}^{{\rm{corr}}}\Vert }_{1}$ does not vanish for finite *J* in contrast to *τ*_4_. However, at $J\approx 0.8$, i.e., well before the critical point, this hierarchy is violated as the four-point correlation ${\Vert {\hat{\rho }}_{4}^{{\rm{corr}}}\Vert }_{1}$ exceeds the three-point correlation ${\Vert {\hat{\rho }}_{3}^{{\rm{corr}}}\Vert }_{1}$. The two-point correlation ${\Vert {\hat{\rho }}_{2}^{{\rm{corr}}}\Vert }_{1}$ is still dominant in this region – this only changes near the critical point. This inversion of the hierarchy, i.e., the dominance of ${\Vert {\hat{\rho }}_{4}^{{\rm{corr}}}\Vert }_{1}$ over ${\Vert {\hat{\rho }}_{3}^{{\rm{corr}}}\Vert }_{1}$ in a region within the symmetric paramagnetic phase, should be relevant for approximation schemes which truncate the hierarchy of correlations at some order^36–42^.

## Bose-Hubbard model

One might suspect that this inversion of the hierarchy is a rather specific result due to the integrability of the model under consideration or may be induced by the fact that deep in the ferromagnetic (broken-symmetry) phase, the three-point correlation vanishes whereas the four-point and two-point correlators approach constant non-zero values (note that an inversion of the two-point and four-point correlations just happens at the critical point). In order to investigate whether the inversion of the hierarchy is a general phenomenon or indeed a peculiar feature of the Ising model, let us consider the other prototypical example for a quantum phase transition^10, 43^, the Bose-Hubbard model

5 $$\hat{H}=-J\sum _{i=1}^{L}\,({\hat{b}}_{i}^{\dagger }{\hat{b}}_{i+1}+{\hat{b}}_{i+1}^{\dagger }{\hat{b}}_{i})+\frac{1}{2}\sum _{i=1}^{L}\,{\hat{b}}_{i}^{\dagger }{\hat{b}}_{i}^{\dagger }{\hat{b}}_{i}{\hat{b}}_{i}$$

that is believed to be non-integrable^44–47^. Here ${\hat{b}}_{i}^{\dagger }$ and ${\hat{b}}_{i}$ are the bosonic creation and annihilation operators at the lattice site *i*. As before, we impose periodic boundary conditions. Note that the hopping rate *J* is dimensionless because we measure it in units of the on-site interaction energy (usually denoted by *U*).

At unit filling $\langle {\hat{n}}_{i}\rangle =1$, there is a quantum phase transition (in the thermodynamic limit *L* → ∞) between the Mott insulator regime where the on-site repulsion dominates (in analogy to the paramagnetic state for the Ising model) and the superfluid phase where the hopping rate *J* dominates (analogously to the ferromagnetic state). Deep in the Mott phase at *J* = 0, the ground state factorizes $|{\rm{\Psi }}\rangle ={\otimes }_{i}{|1\rangle }_{i}$, i.e., it is not entangled. For increasing *J*, on the other hand, we get correlations such as $\langle {\hat{b}}_{i}^{\dagger }{\hat{b}}_{j}\rangle$ which are somewhat analogous to the ferromagnetic correlations $\langle {\hat{\sigma }}_{i}^{z}{\hat{\sigma }}_{j}^{z}\rangle$.

Unfortunately, for the Bose-Hubbard model, entanglement measures in analogy to the concurrence are not yet available. There exist genuine bipartite and multipartite entanglement measures for bosons, but they are known only for special cases such as Gaussian states or pure states (see refs 48, 49 and references therein). Hence, we focus on the reduced density matrices and their correlated parts. We consider a system of finite size (12 bosons on 12 lattice sites) and obtain the ground state numerically for arbitrary *J* by exact diagonalization. This allows to calculate exactly the reduced density matrices. We find that they contain, in contrast to the Ising model, in general more than two non-negligible eigenvalues, i.e., the approximation (2) would not apply here.

**Figure 4:**
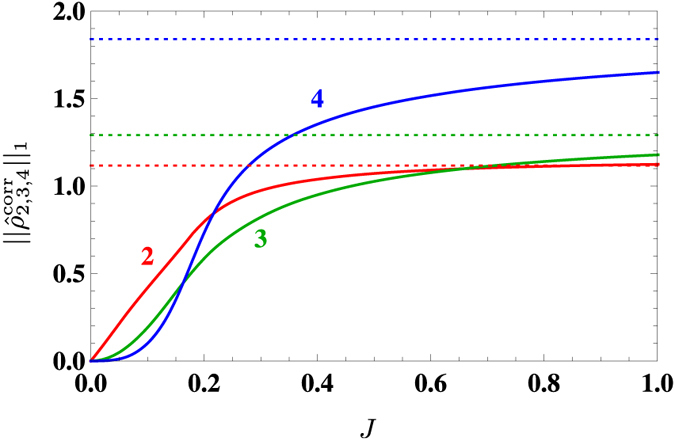
Norms of correlated reduced density operators for two ${\hat{\rho }}_{2}^{{\rm{corr}}}$ (red), three ${\hat{\rho }}_{3}^{{\rm{corr}}}$ (green), and four ${\hat{\rho }}_{4}^{{\rm{corr}}}$ (blue) neighboring sites in the Bose-Hubbard model with 12 particles in 12 lattices sites. The horizontal dotted lines represent the limit of the ideal Bose gas (*J* → ∞). The hierarchy of correlations (first two-point, later three-point and even later four-point correlations) is present for small *J*. The four-point correlations overtake the three-point correlations well before the critical point (here ${J}_{{\rm{crit}}}\approx 0.3$).

In analogy to Fig. 3, we plot the Schatten one-norms of the correlated parts of the reduced density matrices in Fig. 4. We find that – again in contrast to the Ising model – all three curves are monotonically growing and approach finite asymptotic values for *J* → ∞ which correspond to the limit of a free (ideal) Bose gas and can be calculated analytically. Similarly to the Ising model, we find ${\Vert {\hat{\rho }}_{q}^{{\rm{corr}}}\Vert }_{1}\sim {J}^{q-1}$ with *q* = 2, 3, 4 for small *J*, as expected from strong-coupling perturbation theory^24^. This scaling imposes the hierarchy ${\Vert {\hat{\rho }}_{2}^{{\rm{corr}}}\Vert }_{1}\gg {\Vert {\hat{\rho }}_{3}^{{\rm{corr}}}\Vert }_{1}\gg {\Vert {\hat{\rho }}_{4}^{{\rm{corr}}}\Vert }_{1}$ at small values of *J*. However, in analogy to the Ising model, this hierarchy is partially inverted at $J\approx 0.16$ and $J\approx 0.21$, i.e. both well before the critical point is reached (here around ${J}_{{\rm{crit}}}\approx 0.3$, see ref. 50 for a recent review).

## Conclusions and Discussion

For the Ising model (1), we studied the entanglement of two, three, and four neighboring sites in the ground state by means of the approximation (2) based on the dominance of two eigenvalues. In the calculations of the respective tangles for up to four sites, we find a sequential increase of entanglement depth with growing *J* which we call avalanche of entanglement (see Fig. 1). Whereas an interpretation of the results is unambiguous up to three sites, for four sites there are three filter invariants ${\tau }_{4}^{(i)}$, *i* = 1, 2, 3^26^. They give essentially the same result, which together with previous analysis^29, 32^ is providing evidence that a state inside the GHZ class will be the main carrier of entanglement. We conjecture that this avalanche continues until finally pure *L*-partite (GHZ type) entanglement emerges for *J* = ∞. This avalanche might also explain the ln *L* divergence of the entanglement entropy at the critical point, which will be subject of future work.

Using the Schatten one-norms of the correlated reduced density matrices as rigorous upper bounds for the correlations (3), we find that they also yield approximate upper bounds for the corresponding entanglement measures (see Fig. 2). We find a partial inversion of the hierarchy of correlations well before the critical point is reached. Comparison with the Bose-Hubbard model as another prototypical example reveals a qualitatively similar behavior.

This inversion of the hierarchy is relevant for approximation schemes based on truncation^36–42^. One can try to successively improve the accuracy of these approximations by shifting the truncation to higher orders, i.e., by including more correlations. As an example, let us consider a quantity $\langle {\sigma }_{i}^{x}{\sigma }_{j}^{x}{\sigma }_{k}^{x}{\sigma }_{l}^{x}\rangle$ relevant for the Ising model. To lowest order (mean-field limit), one could approximate it via $\langle {\sigma }_{i}^{x}{\sigma }_{j}^{x}{\sigma }_{k}^{x}{\sigma }_{l}^{x}\rangle \approx \langle {\sigma }_{i}^{x}\rangle \langle {\sigma }_{j}^{x}\rangle \langle {\sigma }_{k}^{x}\rangle \langle {\sigma }_{l}^{x}\rangle$, i.e., by neglecting all correlations. As a possible first-order correction, one could include two-point correlations such as ${\langle {\sigma }_{i}^{x}{\sigma }_{j}^{x}\rangle }^{{\rm{corr}}}\langle {\sigma }_{k}^{x}\rangle \langle {\sigma }_{l}^{x}\rangle$. This first-order approximation allows us to derive, e.g., the magnon dispersion relations. While this successive approximation procedure works well for small *J*, we found here that it fails for larger *J*, even well before reaching the critical point.

It might be also interesting to study the possibility of more general approximation schemes such as (2) based on the dominance of two or more eigenvalues of the reduced density operator. In a time-dependent setting one could analyze how this entanglement avalanche is affected by non-adiabatic dynamics during a sweep through the critical point. In fact, the number of non-zero values for the transverse Ising chain has been seen to grow up to four in the case of equal bipartition^51^, such that the approximations applied here could survive further.

## Methods

### Entanglement measures

We consider pure states of *q* spins with two internal degrees of freedom (qubits). For each number of spins *q*, there are corresponding SL-invariant multipartite entanglement measures. In the case of two spins (*q* = 2), the entanglement measure is unique and is given by the concurrence *C*_2_ which coincides with the largest correlation function for pure states^33^.

For three spins, the appropriate entanglement measure is called three-tangle *τ*_3_ (see ref. 13 for the definition). Since we want to compare it to ${\Vert {\hat{\rho }}_{3}^{{\rm{corr}}}\Vert }_{1}$, we consider its square root $\sqrt{{\tau }_{3}}$ because this is also a homogeneous function of degree one with respect to ${\hat{\rho }}_{3}$ (for the entanglement related questions, see refs 30 and 52).

In the case of four spins, there are three in general different entanglement measures (see ref. 28)

6 $${\tau }_{4}^{\mathrm{(1)}}=\sqrt[3]{{ {\mathcal F} }_{1}^{\mathrm{(4)}}},\quad {\tau }_{4}^{\mathrm{(2)}}=\sqrt[4]{{\langle { {\mathcal F} }_{2}^{\mathrm{(4)}}\rangle }_{s}},\quad {\tau }_{4}^{\mathrm{(3)}}=\sqrt[6]{{ {\mathcal F} }_{3}^{\mathrm{(4)}}}.$$

Here the index *s* symbolizes the symmetrization with respect to the symmetric group. They possess the same homogeneous degree as $\sqrt{{\tau }_{3}}$ and *C*_2_ in the density matrix.

### Correlations

We consider the Hamiltonian for a system of *L* lattice sites of the form

7 $$\hat{H}=\sum _{{\ell }_{1}\ne {\ell }_{2}}\,{\hat{H}}_{{\ell }_{1}{\ell }_{2}}+\sum _{\ell }\,{\hat{H}}_{\ell },$$

where ${\hat{H}}_{\ell }$ and ${\hat{H}}_{{\ell }_{1}{\ell }_{2}}$ are local and two-site operators, respectively; the indices label the lattice sites. The state of the whole system can be described by the density operator $\hat{\rho }=|\psi \rangle \langle \psi |$. In order to study parts of the system, we introduce reduced density operators for *q* lattice sites via averaging (partially tracing) over all other sites:

8 $${\hat{\rho }}_{{\ell }_{1}\ldots {\ell }_{q}}={{\rm{Tr}}}_{{\ell }_{q+1}\ldots {\ell }_{L}}\hat{\rho },$$

where all ${\ell }_{1},\ldots ,{\ell }_{L}:\{1,\ldots ,L\}$ are distinct. Information about all possible spatial correlations of the lattice sites ${\ell }_{1}\ldots {\ell }_{q}$ is directly contained in the correlated parts ${\hat{\rho }}_{{\ell }_{1}\ldots {\ell }_{q}}^{{\rm{corr}}}$ of the reduced density operators. For *q* = 2, 3 they are explicitly given by

9 $$\begin{array}{rcl}{\hat{\rho }}_{{\ell }_{1}{\ell }_{2}}^{{\rm{corr}}} & = & {\hat{\rho }}_{{\ell }_{1}{\ell }_{2}}-{\hat{\rho }}_{{\ell }_{1}}{\hat{\rho }}_{{\ell }_{2}}\\ {\hat{\rho }}_{{\ell }_{1}{\ell }_{2}{\ell }_{3}}^{{\rm{corr}}} & = & {\hat{\rho }}_{{\ell }_{1}{\ell }_{2}{\ell }_{3}}-{\hat{\rho }}_{{\ell }_{1}{\ell }_{2}}^{{\rm{corr}}}{\hat{\rho }}_{{\ell }_{3}}-{\hat{\rho }}_{{\ell }_{1}{\ell }_{3}}^{{\rm{corr}}}{\hat{\rho }}_{{\ell }_{2}}-{\hat{\rho }}_{{\ell }_{2}{\ell }_{3}}^{{\rm{corr}}}{\hat{\rho }}_{{\ell }_{1}}-{\hat{\rho }}_{{\ell }_{1}}{\hat{\rho }}_{{\ell }_{2}}{\hat{\rho }}_{{\ell }_{3}}.\end{array}$$

The operators ${\hat{\rho }}_{{\ell }_{1}\ldots {\ell }_{q}}^{{\rm{corr}}}$ are hermitean and their traces vanish: ${\rm{Tr}}{\hat{\rho }}_{{\ell }_{1}\ldots {\ell }_{q}}^{{\rm{corr}}}=0$. They allow to calculate (connected) correlation functions of local operators ${\hat{O}}_{\ell }$ as

10 $${\langle {\hat{O}}_{{\ell }_{1}}\ldots {\hat{O}}_{{\ell }_{q}}\rangle }^{{\rm{corr}}}={\rm{Tr}}({\hat{\rho }}_{{\ell }_{1}\ldots {\ell }_{q}}^{{\rm{corr}}}{\hat{O}}_{{\ell }_{1}}\ldots {\hat{O}}_{{\ell }_{q}}).$$

In order to obtain quantitative estimates of the *q*-point correlations, it is convenient to consider the Schatten *p*-norms

11 $${\Vert {\hat{\rho }}_{{\ell }_{1}\ldots {\ell }_{q}}^{{\rm{corr}}}\Vert }_{p}:=\sqrt[p]{{\rm{Tr}}{|{\hat{\rho }}_{{\ell }_{1}\ldots {\ell }_{q}}^{{\rm{corr}}}|}^{p}}\equiv {(\sum _{I}{|{\lambda }_{{\ell }_{1}\ldots {\ell }_{q}}^{(I)}|}^{p})}^{1/p},$$

where ${\lambda }_{{\ell }_{1}\ldots {\ell }_{q}}^{(I)}$ are the eigenvalues of the correlated density operators ${\hat{\rho }}_{{\ell }_{1}\ldots {\ell }_{q}}^{{\rm{corr}}}$. The Schatten one-norm is also known as the trace norm and the two-norm is often called the Frobenius norm or the Hilbert-Schmidt norm. Note that the quantities ${\Vert {\hat{\rho }}_{{\ell }_{1}\ldots {\ell }_{q}}^{{\rm{corr}}}\Vert }_{p}$ are homogeneous functions of degree one with respect to ${\hat{\rho }}_{{\ell }_{1}\ldots {\ell }_{q}}^{{\rm{corr}}}$.

Assuming that the quantities $|\langle I|{\hat{O}}_{{\ell }_{1}}\ldots {\hat{O}}_{{\ell }_{q}}|I\rangle |$, where |*I*〉 are the eigenstates of the operator ${\hat{\rho }}_{{\ell }_{1}\ldots {\ell }_{q}}^{{\rm{corr}}}$, are bounded by unity (or another finite number), which is the case if the eigenvalues of the operators ${\hat{O}}_{{\ell }_{1}}\ldots {\hat{O}}_{{\ell }_{q}}$ are bounded by unity (spectral norm), it is easy to see that

12 $$\begin{array}{rcl}|{\langle {\hat{O}}_{{\ell }_{1}}\ldots {\hat{O}}_{{\ell }_{q}}\rangle }^{{\rm{corr}}}| & = & |{\rm{Tr}}\{{\hat{O}}_{{\ell }_{1}}\ldots {\hat{O}}_{{\ell }_{q}}{\hat{\rho }}_{{\ell }_{1}\ldots {\ell }_{q}}^{{\rm{corr}}}\}|\\  & = & |\sum _{I}\,{\lambda }_{{\ell }_{1}\ldots {\ell }_{q}}^{(I)}\langle I|{\hat{O}}_{{\ell }_{1}}\ldots {\hat{O}}_{{\ell }_{q}}|I\rangle |\\  & \le  & \sum _{I}\,|{\lambda }_{{\ell }_{1}\ldots {\ell }_{q}}^{(I)}|\\  & = & {\Vert {\hat{\rho }}_{{\ell }_{1}\ldots {\ell }_{q}}^{{\rm{corr}}}\Vert }_{1}.\end{array}$$

Eq. (12) shows that the Schatten one-norm ${\Vert {\hat{\rho }}_{{\ell }_{1}\ldots {\ell }_{q}}^{{\rm{corr}}}\Vert }_{1}$ of the correlated density matrix ${\hat{\rho }}_{{\ell }_{1}\ldots {\ell }_{q}}^{{\rm{corr}}}$ yields an upper bound for the correlations ${\langle {\hat{O}}_{{\ell }_{1}}\ldots {\hat{O}}_{{\ell }_{q}}\rangle }^{{\rm{corr}}}$.

## Electronic supplementary material

Supplementary Material
