# Supplementary material for: Avalanche of entanglement and correlations at quantum phase transitions
Source: Sci Rep. 2017 Jun 16;7:3634. doi: 10.1038/s41598-017-03402-8 (PMC5473896; doi:10.1038/s41598-017-03402-8)
Supplement: Supplementary file 1 — Supplementary Material [file 41598_2017_3402_MOESM1_ESM.pdf]

– *Supplementary Material* –

Avalanche of entanglement and correlations at quantum phase transitions

Konstantin V. Krutitsky, Andreas Osterloh, Ralf Schützhold

Here we report on additional material that supports findings of the main paper.

ENTANGLEMENT VERSUS CORRELATIONS FOR PURE STATES OF QUBITS

One of the main aims of the present work is to study the relation between correlations and entanglement. First, we consider pure states of q spins with two internal degrees of freedom $s = 0, 1$ (qubits):

$$|\psi_q\rangle = \sum_{s_1 \dots s_q} \phi_{s_1 \dots s_q} |s_1 \dots s_q\rangle \ , \ \sum_{s_1 \dots s_q} \left|\phi_{s_1 \dots s_q}\right|^2 = 1 \ . \tag{S1}$$

Definitions of the entanglement measures C_2, τ_3, τ_4 for two, three and four spin-1/2 systems (qubits) are given in Refs. [S1, S2, S3]. It is shown (see Fig. 2 of the main paper) that $\sqrt{\tau_3}$ is almost always bounded from above by $\|\hat{\rho}_3^{\rm corr}\|_1$. In the case of four spins, there are three in general different entanglement measures $\tau_4^{(i)}$, $i = 1, 2, 3$. Looking at the results presented in Fig. S1 for 9×10^5 randomly chosen states $|\psi_4\rangle$, we observe that for the majority of the states $\|\hat{\rho}_4^{\rm corr}\|_1$ gives an upper bound to the four-tangles similarly to the case of three spins just discussed above. Although the values of $\tau_4^{(1)}$, $\tau_4^{(2)}$, $\tau_4^{(3)}$ can differ from each other for a particular state $|\psi_4\rangle$ [S5], this difference is only small here such that the overall picture is basically the same and we display in Fig. S1 only the results for $\tau_4^{(1)}$.

Since the four-tangles as well as the one-norms of the correlated density operators are continuous quantities, there should be neither horizontal nor vertical gaps between individual points in Fig. S1. However, those are visible which is an indication of insufficient statistics. We could not manage to overcome this problem probably due to the large dimension of the corresponding Hilbert space.

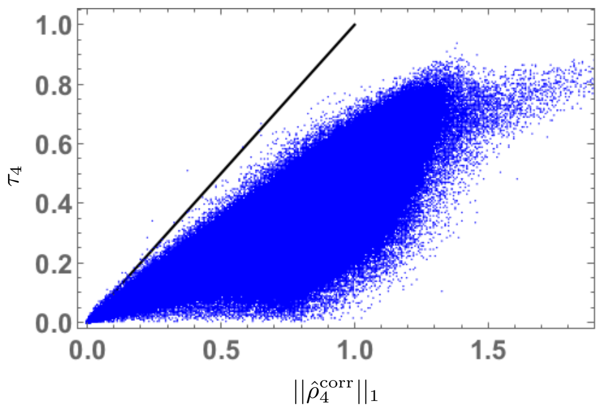

FIG. S1: The four-tangle τ_4 is shown here against $\|\hat{\rho}_4^{\rm corr}\|_1$ for pure states. We have chosen the pure states statistically from the extended Schmidt form [S4]. Each dot in the figure corresponds to a single state. We do not have enough statistics, as can be seen from our viewgraph. Although states for arbitrary value of τ_4 should be there whose value of $\|\hat{\rho}_4^{\rm corr}\|_1$ comes arbitrarily close to the line $\tau_4 = \|\hat{\rho}_4^{\rm corr}\|_1$, we don't see any occurrences at larger values of τ_4.

RANK-TWO APPROXIMATION

The quantum transverse Ising chain is a special case of the transverse XY-chain

$$\hat{H} = -J \sum_{i=1}^{L} \left(\frac{1+\gamma}{2} \hat{\sigma}_i^x \hat{\sigma}_{i+1}^x + \frac{1-\gamma}{2} \hat{\sigma}_i^y \hat{\sigma}_{i+1}^y \right) - \sum_{i=1}^{L} \hat{\sigma}_i^z \ . \tag{S2}$$

For $\gamma \neq 0$ it has a quantum phase transition of the Ising type. The reduced density matrices of two ($\hat{\rho}_2 = \hat{\rho}_{ij}$), three ($\hat{\rho}_3 = \hat{\rho}_{ijk}$), and four ($\hat{\rho}_4 = \hat{\rho}_{ijkl}$) neighboring spins [S6, S7] essentially possess two dominant eigenvalues p_1 and p_2 while the sum of the remaining sub-dominant eigenvalues stays below 2.5%. The second eigenvector interferes strongly with the entanglement of the first, whereas we checked that this is not the case for the third; the remaining error is maximally $p_{I>3} \approx 0.5\%$. Therefore we only consider the case of rank two density matrices and neglect the rest of few percents of weight. What one is left with are the two highest weights p_1 and p_2 of the density matrix. We briefly discuss two ways of taking care of them: 1) take p_1 or 2) take $p_1/(p_1 + p_2)$ as the highest weight of the new rank-two density matrix. Whereas in 1) one assumes that the neglected part is as destructive to the entanglement as the second state is, in 2) the remaining states do not enter the calculation at all. Both treatments lead neither to an upper bound nor to a lower bound to the entanglement.

Details on how the approximations work for the concurrence can be seen in Figs. S2 and S3. The procedure 1) works perfectly for nearest neighbors, where the plots can hardly be distinguished for the transverse Ising chain and also for the transverse XY-chain for J up to the factorising field, where they start to deviate considerably. This is different when considering larger distances, where both curves have similar shapes only around the critical point (see Fig. S3). There, it gives good estimates even for the zeros of the exact concurrence and largely avoids over-estimating the entanglement in the state.

The genuine multipartite entanglement content, i.e., the

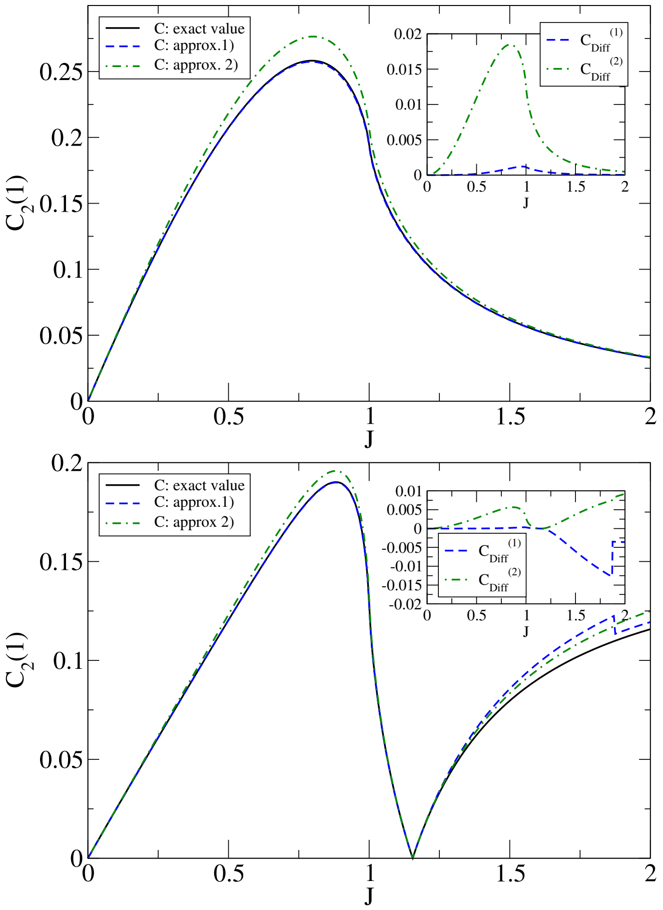

FIG. S2: Top: The exact concurrence $C_2(1)$ is plotted together with the two approximations 1) and 2) (see text) for the transverse Ising model ($\gamma = 1$). It can be seen that approximation 1) (blue dashed curve; almost invisible here) basically coincides with the exact concurrence (black curve). The approximation following scheme 2) (green dash-dotted curve) slightly lies above the exact curve. This is demonstrated in the inset, where the differences $C^{(1)}_{\mathrm{Diff.}} := C - C^{(1)}$ (blue dashed curve) and $C^{(2)}_{\mathrm{Diff.}} := C^{(2)} - C$ (green dash-dotted curve) for approximation schemes 1 and 2 are shown.
Bottom: The same plots as for the transverse Ising chain are shown here for the transverse XY model with anisotropy parameter $\gamma = 0.5$. Here the concurrence $C_2(1)$ is well described by approximation scheme 1) up to the factorising field. Beyond this point, the approximation is still reasonable, but lies above the exact curve.

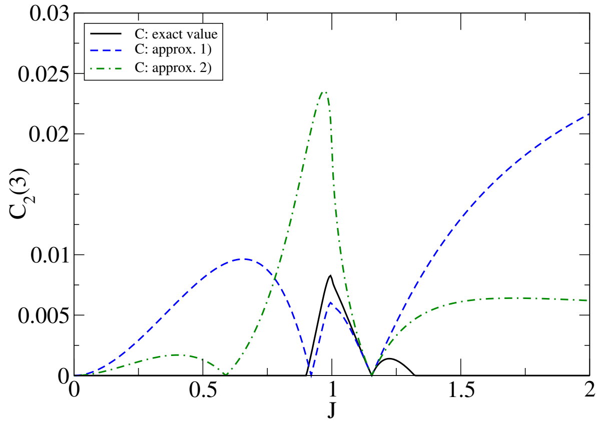

FIG. S3: The concurrence $C_2(3)$ is shown for the transverse XY-model and anisotropy parameter $\gamma = 0.5$. The approximation following scheme 1) is reasonable around the critical point in between the two zeros. Beyond these points it deviates considerably from zero. It however gives a close prediction of the non-trivial zero of $C_2(3)$.

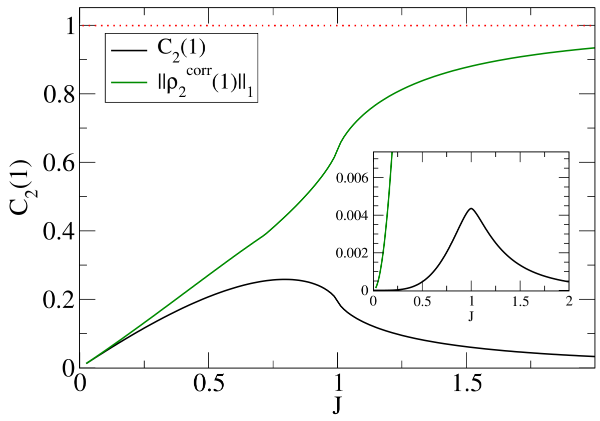

FIG. S4: The concurrence of mixed states $C_2(d)$ and $\|\rho_2^{\mathrm{corr}}(d)\|_1$ for distances $d = 1$ (main figure) and $d = 2$ (inset). For $d = 1$, it is clearly seen that indeed $\|\rho_2^{\mathrm{corr}}(1)\|_1$ as largest correlation function is an upper bound to the corresponding concurrence. For $d = 2$, the concurrence drops to 0.04 at its maximum at the critical point, whereas $\|\rho_2^{\mathrm{corr}}(2)\|_1$ is about roughly the same as $\|\rho_2^{\mathrm{corr}}(1)\|_1$.

three-tangle $\sqrt{\tau_3}$ and the four-tangle, for the nearest-neighboring sites are shown in Fig. 1 of the main article. Numerical calculations show that $\tau_4^{(1)}$, $\tau_4^{(2)}$, and $\tau_4^{(3)}$ for nearest neighbors are roughly the same. Therefore, we do not distinguish between the three four-tangles and drop the upper index. This also indicates that the entanglement would be due to the three- or four-particle GHZ-states, respectively. Such a behaviour goes conform with the expectations for that particular model [S8].

RESULTS FOR THE ISING CHAIN

Now we turn to the ground state of the one-dimensional transverse Ising chain in the thermodynamic limit under periodic boundary conditions and study mixed states of a given number of spins which can be located at different distances from each other. First, we observe that strict equality between the concurrence and largest correlation found for pure states turns into an upper bound for the concurrence of mixed states, i.e., $C_2(\ell_1, \ell_2) \leq ||\hat{\rho}^{\mathrm{corr}}_{\ell_1 \ell_2}||_1$ (see Fig. S4), due to the convex roof involved.

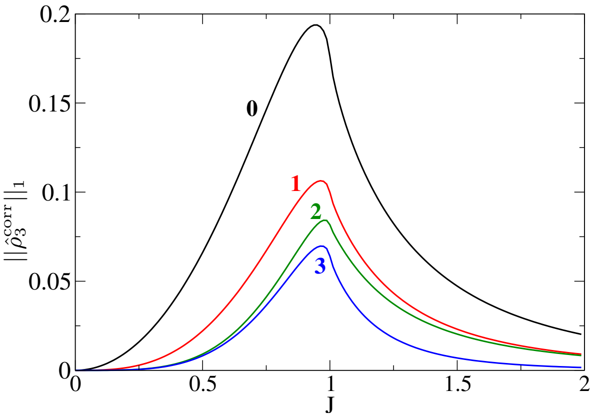

FIG. S5: $\|\hat\rho_3^{\rm corr}(d_1,d_2)\|_1$ is shown for (d_1,d_2) from nearest neighbors $(1,1)$ (black, 0) to $(1,3)$ (green, 2) together with $(2,2)$ (blue, 3). It is not much affected as for the two-site case: if we look at their maximal values, it only shrinks to about one third of that for $\|\hat\rho_3^{\rm corr}(1,1)\|_1$. The maximum is assumed at roughly $J \gtrsim 0.945$ and is slighly moving towards the critical point $J_c = 1$ increasing their distance.

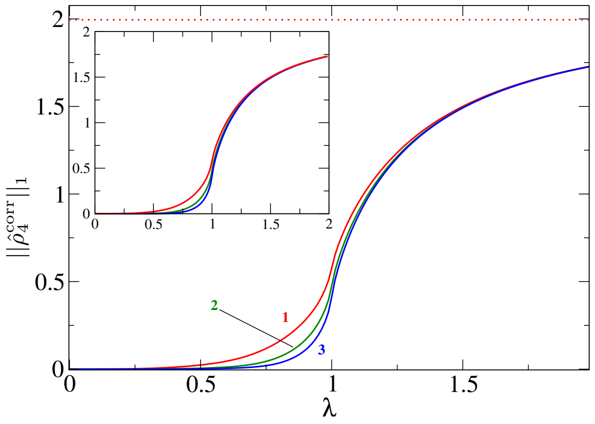

FIG. S6: $\|\hat\rho_4^{\rm corr}\|_1$ is shown for distances corresponding to $(1,d_2,1)$ for $d=1$ (red, 1) to 3 (blue, 3). The effect of growing d seems to be that the curve for d be an upper limit to m with $m<n$. The major change happens around the critical value of $J_c=1$. The inset shows the distances $(2,1,2)$ and $(2,2,2)$ as compared to $(1,1,1)$. There is not much difference noted.

Correlation functions

In what follows, we deal with the ground states of one-dimensional translationally invariant systems. In this case, the density matrices depend only on the distances between the lattices sites. One can always order the site indices such that $\ell_1 < \ell_2 < \cdots < \ell_q$ and we can write $\hat\rho^{\rm corr}_{\ell_1\ldots\ell_q} \equiv \hat\rho_q^{\rm corr}(d_1,\ldots,d_{q-1})$, where $d_i = \ell_{i+1}-\ell_i > 0$ are the corresponding distances. Intuitively, one would expect that (i) the correlations of a fixed number of sites decrease with the distances between the sites and (ii) the correlations for fixed distances decrease with the number of sites. For nearest neighbors, the latter would lead to inequalities

$$||\hat\rho_2^{\rm corr}(1)||_p \gg ||\hat\rho_3^{\rm corr}(1,1)||_p \gg \ldots \qquad \text{(S3)}$$

Our analysis of two completely different models presented below shows that the expectation (i) is always satisfied whereas (ii) does not necessarily hold.

The concurrences $C_2(\ell_1,\ell_2) \equiv C_2(d)$, where $d=|\ell_2-\ell_1|$ are shown together with the 1-norm of $\rho_2^{\rm corr}$ in Fig. S4. Whereas the 1-norm of the correlations has no substantial changes, the concurrence at distance $d=2$ modifies to about 2% of the maximal value for nearest neighbors (see inset).

This qualitatively doesn't change much for the 1-norms considering 3 sites at distances (d_1,d_2) which means that when the first particle is at site ℓ_1 the next is at site ℓ_1+d_1 and the third one at $\ell_1+d_1+d_2$ (hence, the next neighbor reduced density matrix would be $\hat\rho_3(1,1)$). This is shown in Fig. S5, where different distances have been considered for $\|\hat\rho_3^{\rm corr}(d_1,d_2)\|_1$: $(d_1,d_2)=(1,1)$ to $(1,3)$ and $(2,2)$. $\|\hat\rho_3^{\rm corr}(d_1,d_2)\|_1$ decays to about one third of the nearest neighbor situation $\|\hat\rho_3^{\rm corr}(1,1)\|_1$ with a maximum at $J\gtrsim 0.945$ which is moving tinily up to 0.98. One could extract the tendency that the maximum moves for $(1,1)$ to $(1,d_2)$ and from $(1,1)$ to (d_1,d_1) closer to the critical point (where in the latter example we have only considered $(2,2)$ additionally). Observe the astonishingly parallel situation to the two-site case, namely that $\|\hat\rho_3^{\rm corr}(d_1,d_2)\|_1$ doesn't change so drastically with growing distance. This continues to hold for $\|\hat\rho_4^{\rm corr}(d_1,d_2,d_3)\|_1$ (see Fig. S6).

Difference in the norm

We have seen that the 1-norm serves as an upper bound to the correlation functions in the model. We nevertheless studied also the 2-norm, known as the Hibert-Schmidt or Frobenius norm. The result is shown in Fig S7. It is seen that the 2-norm close to the crtitical point $\hat\rho_4^{\rm corr}(1,1,1)$ is still considerably larger than $\hat\rho_3^{\rm corr}(1,1)$; it is however still smaller than $\hat\rho_2^{\rm corr}(1)$ showing only a partial reordering. The corresponding eigenvalues of the matrices $\hat\rho_q^{\rm corr}(1)$, $q=2,3,4$ is shown in Fig. S8.

RESULTS FOR THE BOSE-HUBBARD MODEL

The ground state of the Hamiltonian (4) is obtained numerically for arbitrary J by exact diagonalization in the subspace of the Hilbert space where the total momentum is zero. This allows to calculate exactly the reduced density matrices. In the basis of the occupation numbers $n_1\ldots n_q$, the entries $\langle n_1\ldots n_q|\hat\rho_q(d_1,\ldots,d_{q-1})|n'_1\ldots n'_q\rangle$ do not vanish, provided that $\sum_{i=1}^q n_i = \sum_{i=1}^q n'_i = n_{\rm B} = 0,\ldots,N$. Thus, the reduced density matrices possess a block-diagonal structure and the blocks are labeled by $n_{\rm B}$. The correlated density matrices have a similar structure.

The eigenvalues of the correlated reduced density operators $\hat\rho_q^{\rm corr}(1,\ldots,1)$ are shown in Fig. S9. With the increase of the

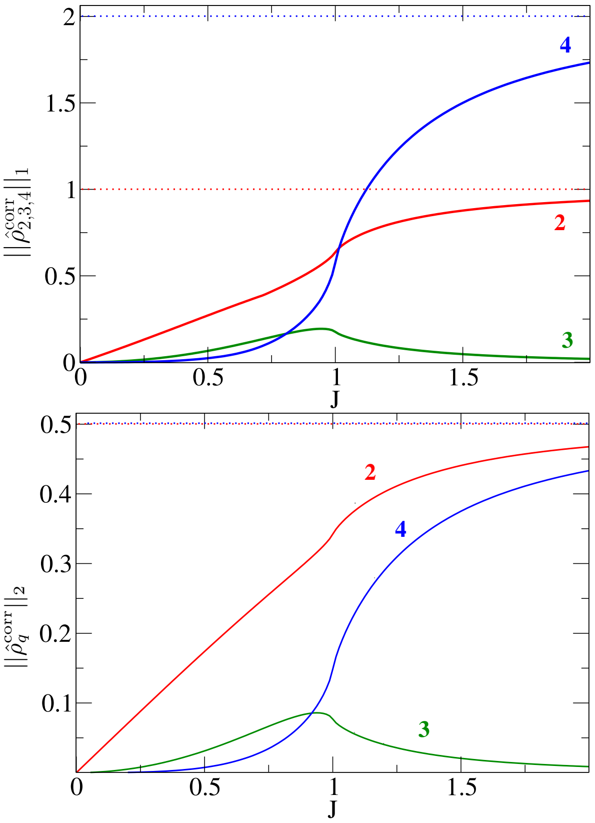

FIG. S7: Norms of correlated reduced density operators $\hat{\rho}_2^{\rm corr}(1)$ (red,2), $\hat{\rho}_3^{\rm corr}(1,1)$ (green,3), $\hat{\rho}_4^{\rm corr}(1,1,1)$ (blue,4) for the ground state of the transverse Ising model. (a) 1-norms. For $J \lesssim 0.8$, $||\hat{\rho}_2^{\rm corr}(1)||_1 > ||\hat{\rho}_3^{\rm corr}(1,1)||_1 > ||\hat{\rho}_4^{\rm corr}(1,1,1)||_1$. For $0.8 \lesssim J \lesssim 1$, $||\hat{\rho}_2^{\rm corr}(1)||_1 > ||\hat{\rho}_4^{\rm corr}(1,1,1)||_1 > ||\hat{\rho}_3^{\rm corr}(1,1)||_1$. For $J \gtrsim 1$, $||\hat{\rho}_4^{\rm corr}(1,1,1)||_1 > ||\hat{\rho}_2^{\rm corr}(1)||_1 > ||\hat{\rho}_3^{\rm corr}(1,1)||_1$. (b) 2-norms. $||\hat{\rho}_2^{\rm corr}(1)||_2$ is always larger than $||\hat{\rho}_3^{\rm corr}(1,1)||_2$ and $||\hat{\rho}_4^{\rm corr}(1,1,1)||_2$. For $J \lesssim 0.9$, $||\hat{\rho}_4^{\rm corr}(1,1,1)||_2 < ||\hat{\rho}_3^{\rm corr}(1,1)||_2$, and for $J \gtrsim 0.9$ we have the opposite.

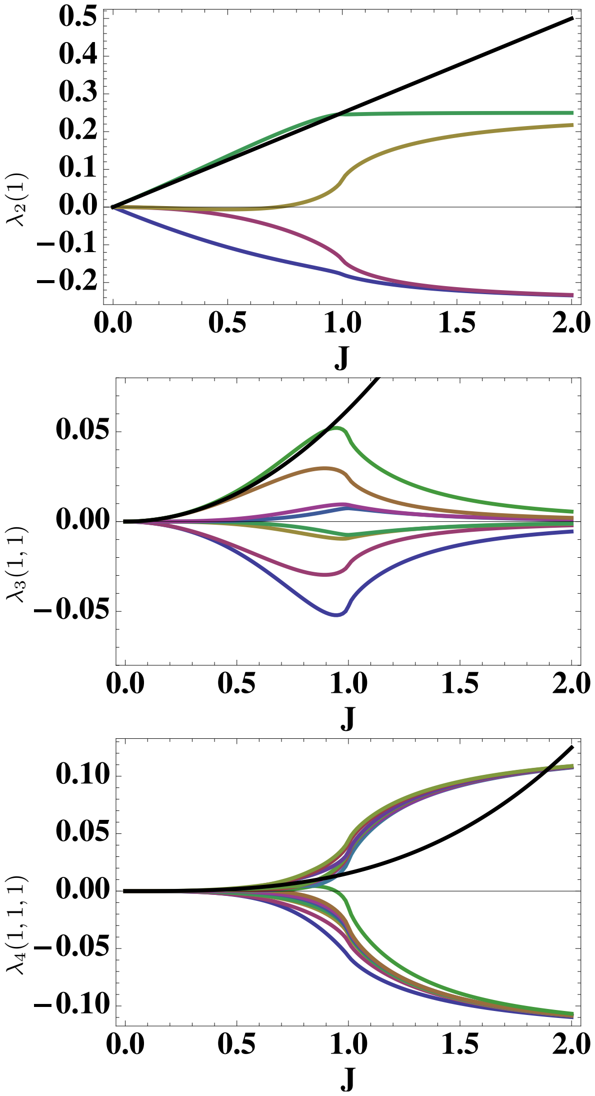

FIG. S8: Eigenvalues of the correlated reduced density operators $\hat{\rho}_2^{\rm corr}(1)$ (a), $\hat{\rho}_3^{\rm corr}(1,1)$ (b), $\hat{\rho}_4^{\rm corr}(1,1,1)$ (c) for the ground state of the transverse Ising model.

number of lattice sites q, the number of nonvanishing eigenvalues grow but their magnitudes decrease. This leads to a qualitatively different behavior of the one- and two-norms that are plotted in Fig. S10. The one-norms $||\hat{\rho}_q^{\rm corr}(1,\ldots,1)||_1$ grow monotonically with the increase of J and tend to finite constant values in the limit $J \to \infty$. For small values of J, we indeed observe the hierarchy (S3) but already at moderate values of J the one-norms for different q become comparable to each other. It is quite surprising that $||\hat{\rho}_4^{\rm corr}(1,1,1)||_1$ becomes quickly larger than $||\hat{\rho}_3^{\rm corr}(1,1)||_1$ and later also larger than $||\hat{\rho}_2^{\rm corr}(1)||_1$. This happens much before the critical point $J_{\rm c}$ of the superfluid–Mott-insulator transition. Hence we observe the same behavior as for the integrable Ising model.

The two-norms $||\hat{\rho}_q^{\rm corr}(1,\ldots,1)||_2$ display completely different behavior because the contribution of small eigenvalues is suppressed. The inequalities (S3) are satisfied for the two-norms at any value of J, although the difference between $q = 2, 3, 4$ is not very large near and above $J_{\rm c}$. The two-norms possess broad maxima and approach their asymptotic values at $J \to \infty$ from above.

If we consider one- and two-norms for fixed q but vary the distances between the sites, we find that both norms decrease with the distance which is demonstrated in Fig. S11 for two sites ($q = 2$). The same was also observed for the transverse Ising model.

The correlated reduced density matrices can be calculated analytically for small values of J, employing the strong-coupling expansion [S9]. In the leading order of J, this gives the following results for their nonvanishing eigenvalues

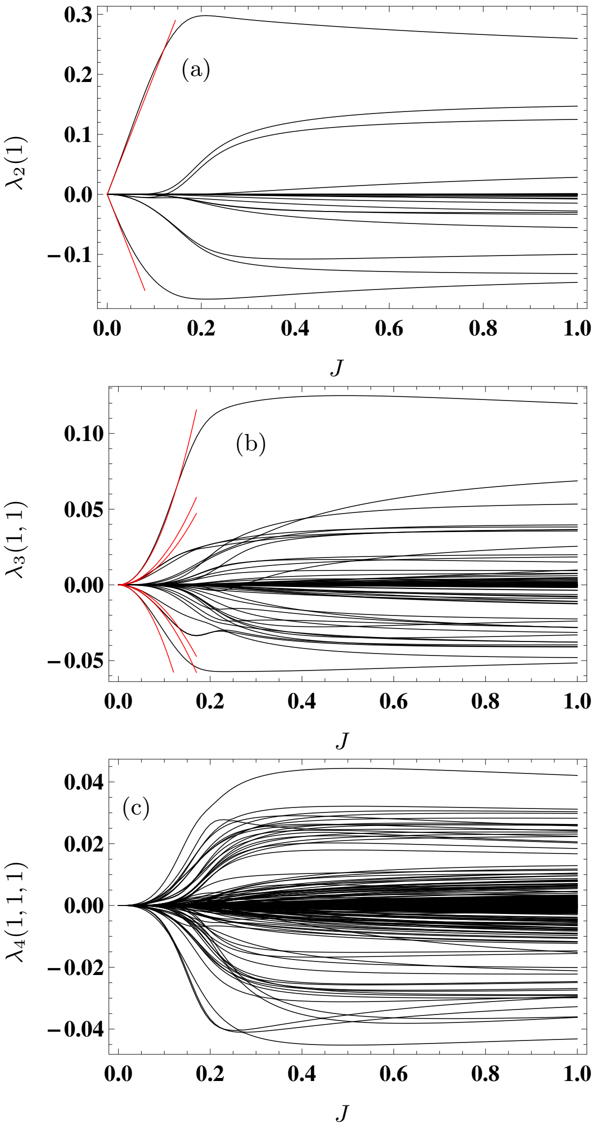

FIG. S9: Eigenvalues of the correlated reduced density operators $\hat{\rho}_2^{\rm corr}(1)$ (a), $\hat{\rho}_3^{\rm corr}(1,1)$ (b), $\hat{\rho}_4^{\rm corr}(1,1,1)$ (c) for the ground state of the Bose-Hubbard model. Black lines – exact diagonalization for $N = L = 12$. Red lines – strong-coupling expansion, see Eqs. (S4).

$\lambda_q^{(I)}(d_1,\ldots,d_{q-1})$:

$$\begin{aligned}
\lambda_2^{(\pm1)}(1) &\approx \pm\sqrt{2n(n+1)}\,J\,, \\
\lambda_2^{(\pm1)}(2) &= \lambda_2^{(\pm2)}(2) \approx \pm n(n+1)\,J^2\,, \\
\lambda_2^{(\pm2)}(2) &\approx \pm(2n+1)\sqrt{2n(n+1)}\,J^2\,, \\
\lambda_3^{(\pm1)}(1,1) &\approx \pm 2n(n+1)\,J^2\,, \\
\lambda_3^{(\pm2)}(1,1) &= \lambda_3^{(\pm3)}(1,1) \approx \pm n(n+1)\,J^2\,, \\
\lambda_3^{(\pm4)}(1,1) &\approx \pm\frac{2}{3}\sqrt{n(n+1)(2n^2+2n-1)}\,J^2\,,
\end{aligned} \tag{S4}$$

where $n = N/L$ is assumed to be an arbitrary integer. Then

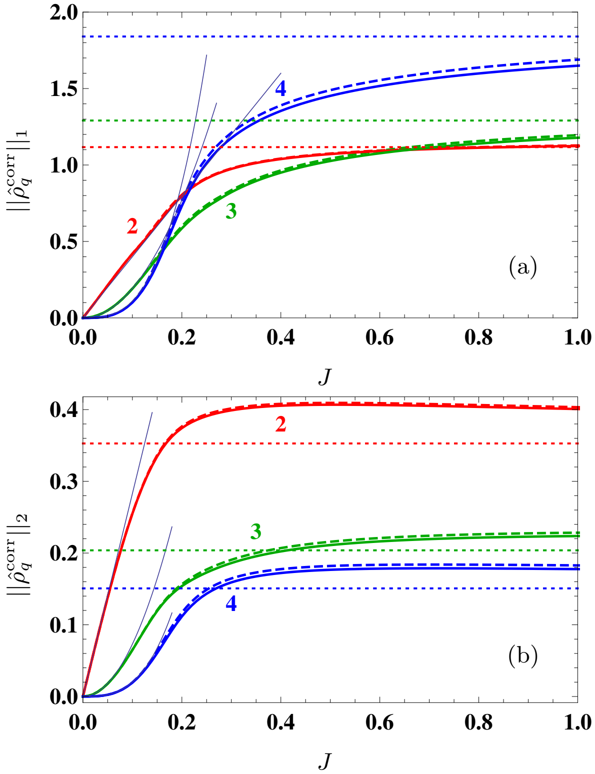

FIG. S10: Norms of correlated reduced density operators $\hat{\rho}_2^{\rm corr}(1)$ (red,2), $\hat{\rho}_3^{\rm corr}(1,1)$ (green,3), $\hat{\rho}_4^{\rm corr}(1,1,1)$ (blue,4) for the ground state of the Bose-Hubbard model. The results of exact diagonalization are shown by dashed curves for $N = L = 9$ and by solid curves for $N = L = 12$. Horizontal dotted lines - the limit of the ideal Bose gas ($J \to \infty$, $N = L = 12$). Thin solid lines – strong-coupling expansion [Eq. (S6)].

for the norms we get

$$\begin{aligned}
||\hat{\rho}_2^{\rm corr}(1)||_1 &\approx 2\sqrt{2n(n+1)}\,J\,, \\
||\hat{\rho}_2^{\rm corr}(1)||_2 &\approx 2\sqrt{n(n+1)}\,J\,, \\
||\hat{\rho}_2^{\rm corr}(2)||_1 &\approx 2\Big[2n(n+1) \\
&\qquad +(2n+1)\sqrt{2n(n+1)}\Big]\,J^2\,, \\
||\hat{\rho}_2^{\rm corr}(2)||_2 &\approx 2\sqrt{n(n+1)(5n^2+5n+1)}\,J^2\,, \\
||\hat{\rho}_3^{\rm corr}(1,1)||_1 &\approx \Big[8n(n+1) \\
&\qquad +\frac{4}{3}\sqrt{n(n+1)(2n^2+2n-1)}\Big]\,J^2\,, \\
||\hat{\rho}_3^{\rm corr}(1,1)||_2 &\approx \frac{2}{3}\left\{n(n+1)\left[31n(n+1)-2\right]\right\}^{1/2} J^2\,.
\end{aligned} \tag{S5}$$

In the special case $n = 1$, this gives

$$\begin{aligned}
&||\hat{\rho}_2^{\rm corr}(1)||_1 \approx 4J\,, \quad ||\hat{\rho}_2^{\rm corr}(1)||_2 \approx 2.82J\,, \\
&||\hat{\rho}_3^{\rm corr}(1,1)||_1 \approx 19.266J^2\,, \quad ||\hat{\rho}_3^{\rm corr}(1,1)||_2 \approx 7.3J^2\,,
\end{aligned} \tag{S6}$$

which is in excellent agreement with our numerical calculations (see Fig. S10). The norms of the correlated reduced

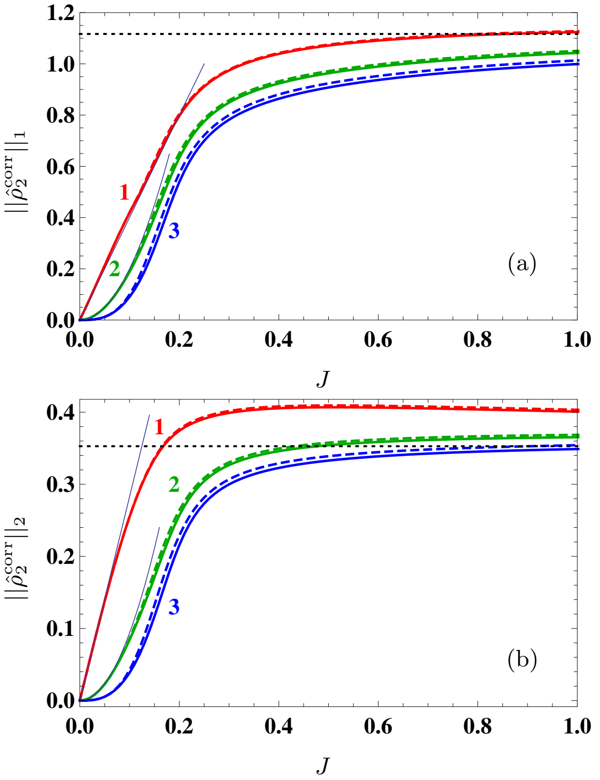

FIG. S11: Norms of correlated reduced density operators $\hat{\rho}_2^{\rm corr}(1)$ (red,1), $\hat{\rho}_2^{\rm corr}(2)$ (green,2), $\hat{\rho}_2^{\rm corr}(3)$ (blue,3) for the ground state of the Bose-Hubbard model. The results of exact diagonalization are shown by dashed curves for $N = L = 9$ and by solid curves for $N = L = 12$. Horizontal dotted lines - the limit of the ideal Bose gas ($J \to \infty$, $N = L = 12$). Thin solid lines – strong-coupling expansion [Eq. (S5)].

density operators grow with n.

In the limit of the ideal Bose gas ($J \to \infty$), the entries of the reduced density matrices depend only on the number of sites q but not on the distances between those:

$$\langle n_1 \dots n_q|\hat{\rho}_q|n_1' \dots n_q'\rangle = \frac{N!}{(N-n_{\rm B})!n_{\rm B}!}\left(\frac{n_{\rm B}!}{n_1!\dots n_q!}\right)^{1/2} \times \left(\frac{n_{\rm B}!}{n_1'!\dots n_q'!}\right)^{1/2}\left(1-\frac{q}{L}\right)^{N-n_{\rm B}}\left(\frac{1}{L}\right)^{n_{\rm B}} . \quad \text{(S7)}$$

Eq. (S7) leads to rather simple expressions for the 2-norms in the thermodynamic limit

$$\begin{aligned} ||\hat{\rho}_2^{\rm corr}||_2 &= \left[I_0(4\langle\hat{n}\rangle) - I_0^2(2\langle\hat{n}\rangle)\right]^{1/2} e^{-2\langle\hat{n}\rangle} , \\ ||\hat{\rho}_3^{\rm corr}||_2 &= \left[I_0(6\langle\hat{n}\rangle) - 3I_0(2\langle\hat{n}\rangle)I_0(4\langle\hat{n}\rangle) \right. \\ &\quad \left. + 2I_0^3(2\langle\hat{n}\rangle)\right]^{1/2} e^{-3\langle\hat{n}\rangle} , \end{aligned} \quad \text{(S8)}$$

where $\langle\hat{n}\rangle = N/L$ is not necessarily an integer and $I_0(x)$ is the modified Bessel function of the first kind. For $\langle\hat{n}\rangle = 1$, this yields $||\hat{\rho}_2^{\rm corr}||_2 \approx 0.334$, $||\hat{\rho}_3^{\rm corr}||_2 \approx 0.184$. These values are slightly lower than those shown in Fig. S10(b) indicated by the horizontal dotted lines, which is a manifestation of the finite-size effects. For large filling factors $\langle\hat{n}\rangle$, the 2-norms tend to zero as

$$||\hat{\rho}_2^{\rm corr}||_2 \approx \frac{1}{\sqrt{8\pi\langle\hat{n}\rangle}} , \quad ||\hat{\rho}_3^{\rm corr}||_2 \approx \frac{1}{\sqrt{12\pi\langle\hat{n}\rangle}} , \quad \text{(S9)}$$

which follows from the asymptotics of $I_0(x)$ for large x.

[S1] W. K. Wootters, Phys. Rev. Lett. **80**, 2245 (1998).
[S2] V. Coffman, J. Kundu, and W. K. Wootters, Phys. Rev. A **61**, 052306 (2000).
[S3] D. Ž. Đoković and A. Osterloh, J. Math. Phys. **50**, 033509 (2009).
[S4] H. A. Carteret, A. Higuchi, and A. Sudbery, J. Math. Phys. **41**, 7932 (2000).
[S5] A. Osterloh and J. Siewert, Int. J. Quant. Inf. **4**, 531 (2006).
[S6] P. Pfeuty, Ann.Phys. **57**, 79 (1970).
[S7] M. Hofmann, A. Osterloh, and O. Gühne, Phys. Rev. B **89**, 134101 (2014).
[S8] A. Osterloh, Phys. Rev. A **93**, 052322 (2016).
[S9] J. K. Freericks and H. Monien, Phys. Rev. B **53**, 2691 (1996).
